# Prevalence, implementation, and barriers of sodium bicarbonate supplementation in team sports

**DOI:** 10.5114/biolsport.2026.156235

**Published:** 2026-06-24

**Authors:** William H. Gurton, Lewis A. Gough, Anthony Lynn, Mayur K. Ranchordas

**Affiliations:** 1 Sport & Physical Activity Research Centre, Health Research Institute, Sheffield Hallam University, UK; 2 Human Performance & Health Research Group, Centre for Life & Sport Sciences, Birmingham City University, UK; 3 Food and Nutrition Subject Group, Sheffield Hallam University, UK; 4 Advanced Wellbeing Research Centre, Sheffield, UK

**Keywords:** Ergogenic Aids, Nutrition, Practitioners, Performance, Survey

## Abstract

Sodium bicarbonate (SB) ingestion has empirical evidence supporting its efficacy for improving team sport performance. It is currently unclear, however, if SB is incorporated into nutritional regimes by practitioners. This study aimed to investigate existing prevalence, implementation, and barriers of SB supplementation in team sports. A cross-sectional, observational design was employed. In total, 66 practitioners (professional experience: 7 ± 5 years) based in UK or USA responded to an online survey. Most practitioners (n = 60; 91%) were aware of SB being used as an ergogenic aid, which was predominately attributed to reputable sources (e.g., scientific literature: n = 35; 58%). Prevalence rates were low (n = 20; 33%), with a significant difference between the frequency of responses for practitioners who use SB (*χ*^2^ = 6.667, p = 0.010). Concerns about gastrointestinal (GI) side-effects after SB was the most common barrier to use (n = 27; 65%), with a significant difference between the frequency of responses (*χ*^2^ = 43.875, p < 0.001) compared to factors like budget. SB is frequently prescribed as a 0.2–0.3 g//kg body mass dose (n = 12, 60%), whilst traditional administration approaches are used more commonly (n = 10; 50%) than novel forms such as topical muscle lotion (n = 4; 20%). A translational gap exists between science and real-world practice, with negative connotations about GI side-effects preventing UK and USA based practitioners from using SB with team sports. Future work should educate practitioners on enteric and hydrogel mini-tablet designed SB that alleviate GI side-effects.

## INTRODUCTION

Sodium bicarbonate (SB) is one of only a few ergogenic aids that has sufficient evidence supporting its efficacy for improving team sport performance [1–4]. Ingestion of 0.2–0.4 g/kg body mass (BM) SB has improved key predictors of success in team sports such as intermittent shuttle run performance [1, 3], repeated sprint times [3–5], vertical jump height [6] and skilled task ability [2] throughout isolated testing batteries and simulated match play exercise protocols. The mechanisms by which SB exerts its ergogenic effects are most likely underpinned by increases in exogenous bicarbonate ion (HCO_3_^−^) concentration (~5–6 mmol/L) [7] or favorable movement of isolated (i.e., potassium) or collective ionic movements (i.e., the strong ion difference) [8, 9]. Despite theoretical benefits, however, very little is known as to whether SB supplementation is used in teams sports by practitioners to support sports performance and/or recovery.

A considerable number of studies have examined dietary supplement use amongst individual and team sport athletes [10]. One ergogenic aid widely used in team sports is caffeine, with Tallis et al. [11] showing that the supplement was prescribed as a means for improving performance by 97% of a sample of professional UK soccer clubs. On the other hand, the frequency of athletes and/or practitioners regularly using SB appears to be low [12, 13]. Early research by Schröder et al. [13] found that SB was not considered a worthwhile ergogenic aid by professional basketball players. In a cohort of elite Spanish individual and team sport athletes, SB was also not reported as one of the sixteen most frequently used supplements [12]. More recently, Newbury et al. [14] observed that 36% of national level swimmers from a high-performance UK club used SB, but it is difficult to translate these findings to team sports, not least as individual sport athletes typically report a higher use of supplements [13, 15]. Collectively, these studies failed to directly investigate the prevalence and practices of SB as an ergogenic strategy in team sports, meaning it is not possible to establish to what extent the supplement is incorporated within nutritional regimes.

Existing research is also yet to systematically evaluate prominent reasons for why athletes and practitioners alike do not use SB, regardless of whether it improves exercise performance. One explanation likely relates to gastrointestinal (GI) discomfort. It has been widely established that ingestion of SB in ergogenic doses causes GI side-effects such as stomach ache and diarrhoea [16, 17]. Durkalec-Michalski et al. [16] showed an inverse relationship between GI distress and anaerobic power (*r* = -0.674, *p* = 0.002), therefore administration strategies that alleviate side-effects after SB ingestion are crucial for refining translation of scientific literature into real-world practice. Traditional strategies including smaller dosing loads (e.g., 0.15–0.2 g/kg BM) [17, 18] and progressive-chronic protocols (e.g., increments from 0.05 up to 0.2 g/kg BM) [2, 19] have been examined, but GI distress can sometimes still occur. Recently, novel SB forms like enteric-coated capsules [20, 21] and hydrogel mini-tablets [22] have emerged. These have also been shown to reduce side-effects and improve performance [20–22], although their translation to the real-world is only just gaining traction. Another prospect is a topical SB cream called PR Lotion. This eliminates the risk of GI distress, but its efficacy as a ergogenic aid has been questioned [3, 4, 23]. Whilst additional research is needed that compares the performance enhancing effects of novel SB forms to traditional ingestion approaches, they offer an attractive alternative for those concerned about GI side-effects [23–25]. It is vital to explore current understanding and perception of novel SB forms to attain practitioners’ opinions about their suitability in team sports and determine whether evidence-based practices are being followed.

Therefore, the purpose of the present study was two-fold. Primarily, it aimed to investigate the use of SB supplementation as an ergogenic strategy in team sports. It was decided that the focus would be on practitioners, rather than the athletes themselves, as practitioners are the major influencing factor for the design and prescription of nutritional practices in team sports [26]. In the first instance, our study sought to explore SB supplementation prevalence, rationale for/against use, administration approaches and timing. Thereafter, this study aimed to gather practitioners’ opinions about three novel SB forms—enteric-coated, hydrogel mini-tablets, and topical muscle lotion—and obtain practical insight to inform future research direction.

## MATERIALS AND METHODS

### Study design

A cross-sectional, observational design was employed for this study. An electronic survey (Qualtrics, Provo, USA) was used to investigate the prevalence and understanding of SB supplementation as an ergogenic strategy in team sports. This survey was distributed to UK and USA based team sport practitioners predominately through professional networks via WhatsApp chats and word-of-mouth, and targeted social media recruitment (e.g., direct messages on LinkedIn). Data collection took place from November 2023 to February 2025. The study was approved by the Institutional Ethics Committee (ER58230829), and all participants signed an electronic informed consent before responding to the survey.

### Participants

Given the exploratory nature of this research, there were no data available for sample size estimation. Therefore, our target sample size was based off previous studies examining the prevalence and understanding of ergogenic aids in practitioners [11, 27]. In total, 70 practitioners working with team sports provided consent to take part in the study. Data were obtained from 66 practitioners (41 males, 25 females; age: 36 ± 10 years; professional experience: 7 ± 5 years) as some did not finish the online survey. Inclusion criteria required participants to be ≥ 18 years old and have ≥ 6 months experience working with team sports. Practitioners based in UK or USA were targeted for their sampling convenience (e.g., professional networks of the research team) and because the majority of scientific work on novel SB forms originates from these countries [3, 4, 20, 22, 25, 28]. The practitioners were typically nutritionists (*n* = 50), but some were sport scientists (*n* = 11), or strength & conditioning coaches (*n* = 5). Team sports were defined as a sport comprising repeated bouts of high-intensity exercise involving a collective of individuals competing together [29]. Practitioners were asked to list what team sports they had regularly worked with during the previous 6 months (Fig. 1A) and whether they had worked with youth/academy, collegiate, semi-professional or professional athletes (Fig. 1B). They most commonly reported working with male team sports (*n* = 39), although some were involved with female athletes, either exclusively (*n* = 3) or additionally (*n* = 24).

**FIG. 1:**
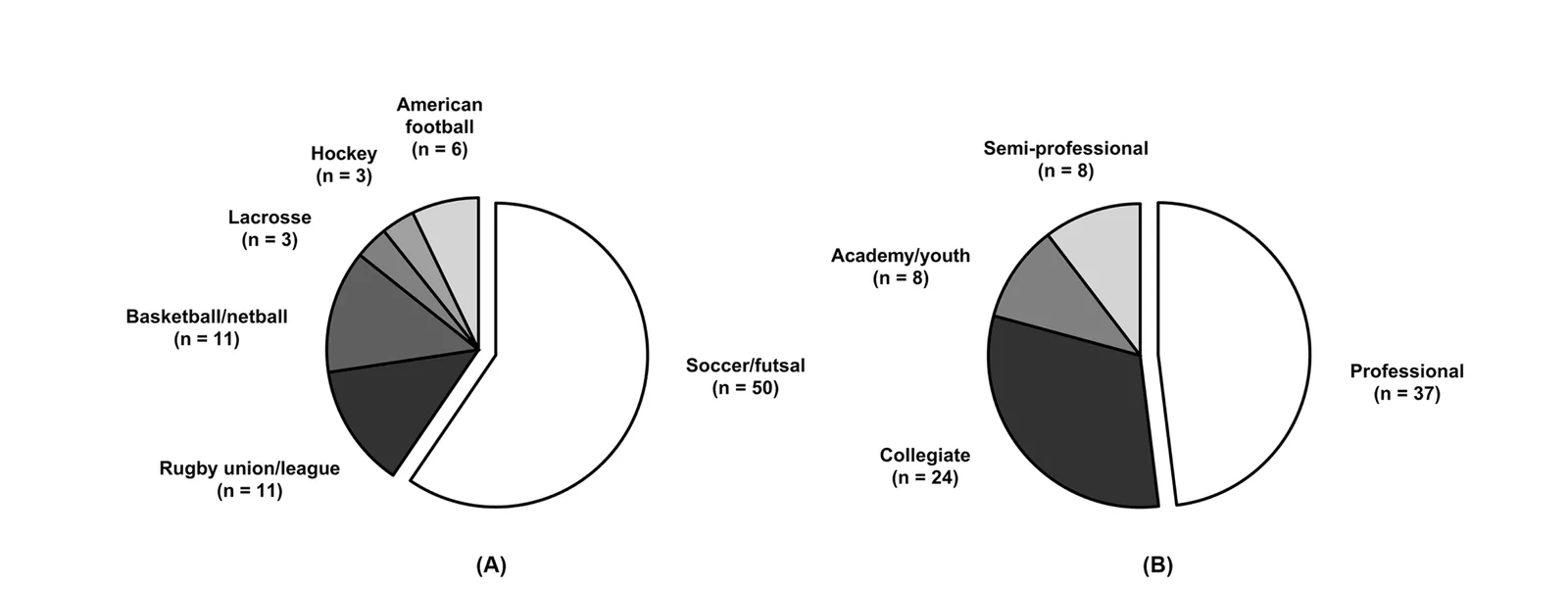
Team sport (A) and training status of athlete (B) that practitioners responding to the survey worked with on a regular basis.

### Questionnaire

The questionnaire was developed by our research team who have extensive experience of conducting SB research and working with team sport athletes. Questionnaire design and flow was based off previous surveys investigating the prevalence and understanding of ergogenic aids [11, 30, 31]. Prior to distributing the final version of the survey, feedback was sought regarding its validity and usability from a small-scale pilot sample (*n* = 10) of subject experts and local team sport practitioners between July and November 2023. In response to their answers and feedback, minor wording (e.g., clarity of questions) and structural changes (e.g., order of questions) were made to the questionnaire to make it as clear and user friendly as possible to prevent practitioners from misinterpreting any of the questions.

The final version of the survey consisted of a maximum of 27 questions split across 3 separate sections: *part 1*, participant demographics; *part 2*, understanding and prevalence of SB as an ergogenic aid; *part 3*, novel SB forms and future research. Following the demographics section, practitioners were asked whether they were aware of SB being used as a sport supplement and if they had prescribed it to team sport athletes. Based on their answers, they were either presented with follow-up questions on understanding and prevalence of SB as an ergogenic aid (e.g., rationale for/against use, dosage, administration approach, timing), or automatically directed to section 3 using Qualtrics skip-display logic. Question types were single choice (i.e., most relevant), multiple selection (i.e., all that are relevant) and open-ended (i.e., other). To remove any ambiguity and gain a clear insight into SB practices employed, practitioners were asked to choose from prespecified options when answering questions about dosage (“ < 0.2 g/kg BM”; “0.2–0.3 g/kg BM”; “ > 0.3 g/kg BM”), administration approach (“solution”; “pre-filled capsules”; “hydrogel system”; “muscle lotion”) and timing (“during training”; “before a match”; “at half-time”; “post-match/recovery”). Opinions relating to use of novel SB forms (enteric-coated capsules, hydrogel mini-tablets, and topical muscle lotion) were nested under preliminary questions asking whether practitioners were aware of them—a follow up brief was provided for those who had not heard of one. Lastly, practitioners were asked to offer some applied insight regarding the focus of future research. To ensure data collected remained worthwhile and robust, prespecified options (“real-world studies”; “recovery interventions”; “hydration application”; “unsure”) were provided based off our understanding of scientific literature and anticipated future research trends.

### Statistical analysis

All data were exported from Qualtrics before being collated on a digital workbook, checked and analyzed using Excel (Microsoft, Washington, USA) and SPSS version 26 (IBM, New York, USA). Figures were created using graphical software (GraphPad Prism, Boston, USA). Descriptive data are reported as response frequencies and percentages (%). Depending on question type, % data was calculated as overall proportion (single choice questions, e.g., preferred dosage), or proportion of practitioners (multiple choice questions, e.g., barriers to using SB). Goodness of fit chi-square (*χ*^2^) was used to assess differences in the frequency of responses amongst practitioners for their awareness, understanding and application of SB. Cramer’s V statistic (*V*) was reported as the effect size and interpreted as small (0.1–0.3), medium (0.3–0.5), and large (> 0.5). Statistical significance was set at *p* < 0.05.

## RESULTS

### Awareness and prevalence rate

There was a significant difference in the frequency of practitioners who were aware of SB being used as an ergogenic aid (*χ*^2^ = 44.182, *p* < 0.001, *V* = 0.82), with 91% (*n* = 60) stating that they had heard of the SB compared with 9% (*n* = 6) who had not. The practitioners’ awareness of SB came from a variety of sources: scientific literature (*n* = 35), other practitioners and/or staff (*n* = 16), higher education (*n* = 8), and social media/advertising (*n* = 1) (Table 1). There was a significant difference in the frequency of these responses (*χ*^2^ = 43.067, *p* < 0.001, *V* = 0.49). Out of the practitioners who were aware of SB, 87% (*n* = 52) suggested that they believe it could improve the performance of team sport athletes, but only 33% (*n* = 20) used SB within their own practice. In contrast, 67% (*n* = 40) reported never previously prescribing SB (*n* = 40), such that the frequency of responses compared to those who did was significantly different (*χ*^2^ = 6.667, *p* = 0.010, *V* = 0.33).

**TABLE 1:** Information sources cited by practitioners for their awareness of sodium bicarbonate

Information source	Proportion (%)
Scientific literature	58
Other practitioners/staff	27
Higher education (BSc, MSc etc.)	13
Social media/advertising	2
Other (…)	0

*Note:* Practitioners (*n* = 60) asked to select the most appropriate. Significant difference in the frequency of responses (*p* < 0.05).

### Reasons for and against use

Out of the 20 practitioners who used SB, almost all of them (*n* = 16) reported that they prescribed SB to improve team sport-specific exercise performance. Although less common, other pre-specified reasons for using SB included reducing perception of fatigue (*n* = 6), accelerating recovery after a match or training (*n* = 4), and improving fluid retention (*n* = 4) (Fig. 2A). The frequency of responses were significantly different from each other (*χ*^2^ = 13.200, *p* = 0.004, *V* = 0.38).

**FIG. 2:**
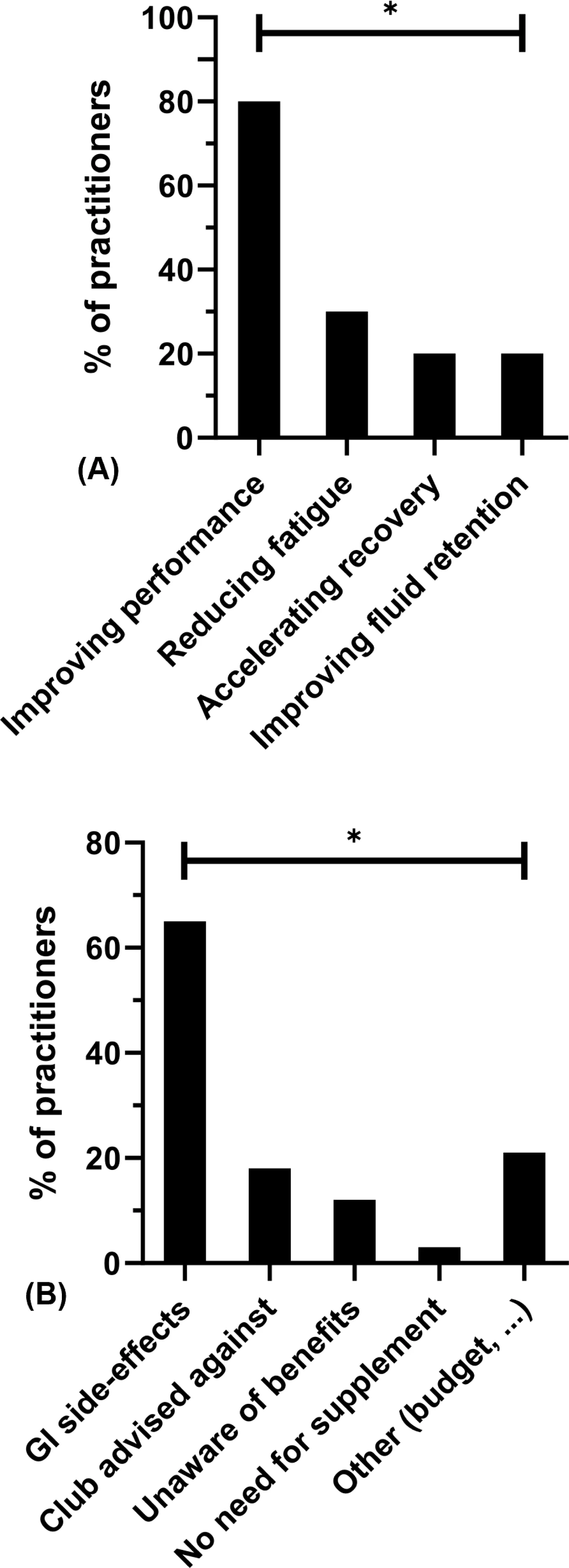
Reasons given by practitioners for (A) and against (B) using SB supplementation in team sports. Respondents asked to select all relevant options, so data presented as % of practitioners. Symbol (*) denotes significant difference in frequency of responses between options (p < 0.05).

Of the remaining 40 practitioners who did not use SB, despite being aware of the supplement, a variety of reasons were cited that prevented them from prescribing SB to their athletes. There was a significant difference in the frequency of responses (*χ*^2^ = 43.875, *p* < 0.001, *V* = 0.49), with the largest proportion of practitioners suggesting that they were concerned about GI side-effects (*n* = 27). Other barriers to using SB included that the club had advised against it (*n* = 6), being unaware of the benefits (*n* = 4), and the belief that there is no need for the supplement (*n* = 1). Some practitioners expanded upon these pre-specified reasons by stating that poor tolerance/taste (*n* = 5), impracticality/logistics (*n* = 2), and budget limitations (*n* = 3) also contribute to why they do not use SB (Fig. 2B).

### Supplementation practices

SB is most commonly administered in a 0.2–0.3 g/kg BM dosage (*n* = 12), but some practitioners prescribe a lower dose < 0.2 g/kg BM (*n* = 7). Only one practitioner reported prescribing SB as a dose > 0.3 g/kg BM (Fig. 3A). There was a significant difference in frequency of responses (*χ*^2^ = 9.100, *p* = 0.011, *V* = 0.48). In terms of administration approaches, SB is dissolved in liquid most commonly (*n* = 10). Other approaches included pre-filled capsules (*n* = 4), topical muscle lotion (*n* = 4), and hydrogel mini-tablets (*n* = 2) (Fig. 3B). The frequency of responses were similar (*χ*^2^ = 7.200, *p* = 0.066, *V* = 0.35). Reasons for why practitioners chose a certain administration approach typically related to reducing GI side-effects (*n* = 9) or improving palatability/tolerance (*n* = 5). In some scenarios, however, practitioners were not aware of alternatives (*n* = 3) or had been advised by another member of staff (*n* = 3) (Table 2). The frequency of responses were similar (*χ*^2^ = 4.800, *p* = 0.187, *V* = 0.28). With regards to supplement timing, almost all of the practitioners (*n* = 17) suggested that they administered SB before a match. There was a significant difference in the frequency of responses (*χ*^2^ = 22.333, *p* < 0.001, *V* = 0.53), with administration at half-time (*n* = 2), during training (*n* = 6), or after a match (*n* = 2) reported considerably less frequently (Table 3).

**TABLE 2:** Reasons given by practitioners for why they opt for their preferred sodium bicarbonate ingestion approach

Reason	Proportion (%)
Reducing GI side-effects	45
Improving palatability/tolerance	25
Unaware of alternatives	15
Advised by club/staff members	15
Other (…)	0

*Note:* Practitioners (*n* = 20) asked to select the most appropriate. Frequency of responses similar between options (*p* > 0.05).

**TABLE 3:** Timing of sodium bicarbonate supplementation by practitioners

Supplement timing	% of practitioners
Pre-match	85
At half-time	10
During training	30
After a match	10
Other (…)	0

*Note:* Practitioners (*n* = 20) asked to select all that were relevant. Significant difference in the frequency of responses (*p* < 0.05).

**FIG. 3:**
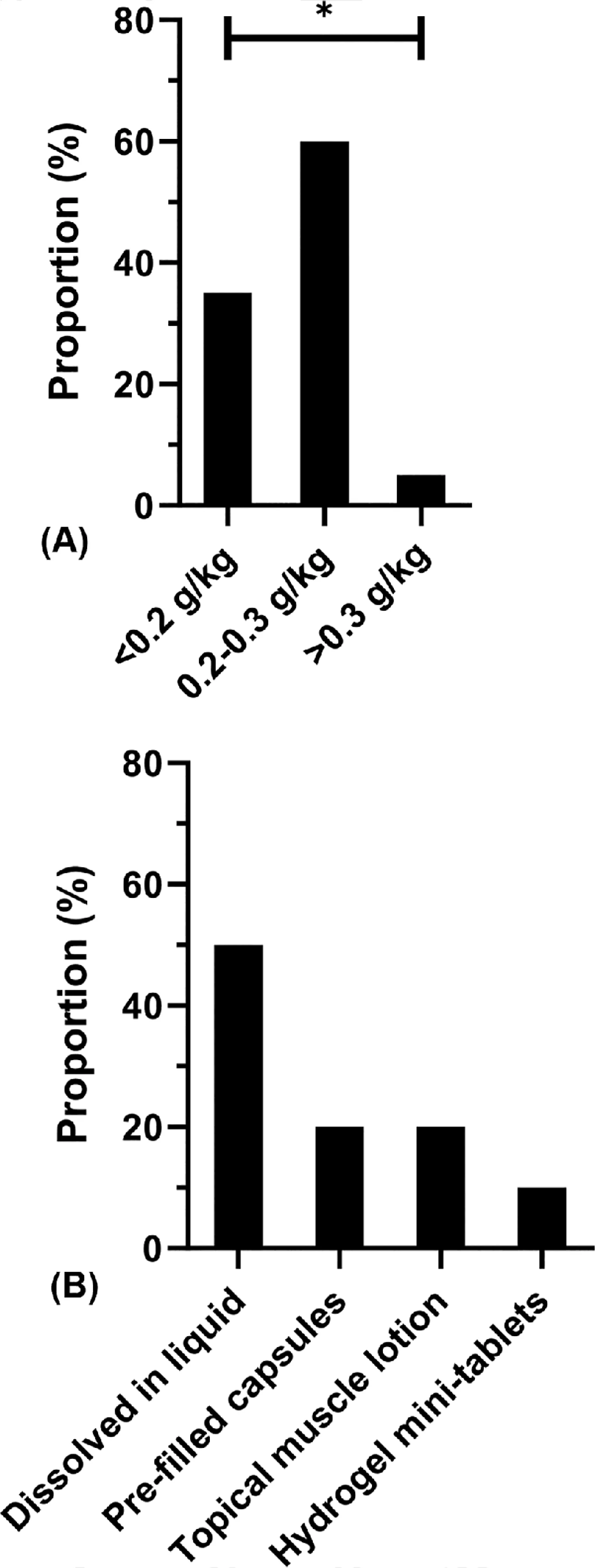
Dosage (A) and administration approach (B) adopted by practitioners who use SB with team sport athletes. Respondents asked to select the most appropriate option, so overall proportion is represented as a % of the cohort (n = 20). Symbol (*) denotes significant difference in frequency of responses between options (p < 0.05).

### Novel SB forms and future research

Out of all the practitioners, 23% (*n* = 15) had not heard of any of the novel SB forms. The frequency of those who were aware of each form was not significantly different (*χ*^2^ = 2.435, *p* = 0.296, *V* = 0.12), with a similar number of practitioners stating an awareness of topical muscle lotion (*n* = 36), hydrogel mini-tablets (*n* = 32) and enteric-coated capsules (*n* = 24). Practitioners reported they would be more likely to use topical muscle lotion (*n* = 45) than enteric-coated capsules (*n* = 36) or hydrogel mini-tablets (*n* = 22), such that the frequency of responses was significantly different between SB forms (*χ*^2^ = 7.825, *p* = 0.020, *V* = 0.19). Lastly, most practitioners suggested further work should be conducted on the effect of SB during real-world studies that examine competitive match scenarios (*n* = 35) or recovery (*n* = 23). There were also some who were unsure about the direction of future research (*n* = 6) and others (*n* = 2) who believed it should investigate the application of SB as a hydration aid.

## DISCUSSION

The present study is the first to examine the prevalence, implementation, and barriers of SB supplementation as an ergogenic strategy in team sports. Our data from UK or USA based practitioners working with a range of team sports across youth/academy, collegiate, semi-professional, or professional levels shows that SB is not currently widely used, despite good awareness of the supplement—predominately from reputable sources like scientific literature—and the belief that it may improve performance. The practitioners that use SB typically administer it prior to matches and as 0.2–0.3 g/kg BM doses. The most common reason why practitioners do not use SB with athletes are concerns about GI side-effects. Understanding and practices of SB administration strategies able to alleviate GI distress is lacking, which could be due to practitioners’ poor knowledge base beyond surface level, or their unwillingness to trial alternative traditional strategies. Awareness of novel SB forms (enteric-coated, hydrogel mini-tablets, and topical muscle lotion) also seems to be limited, although this might be attributed to their relatively recent emergence. Ultimately, the fresh perspectives presented throughout this study provide a nuanced explanation for why the translation of SB as an ergogenic aid in UK and USA team sports is poor despite empirical evidence supporting its efficacy. Future work should aim to educate practitioners about SB administration strategies that alleviate GI side-effects and compare the effectiveness of traditional and novel SB forms in real-world team sport settings to refine evidence-based practices.

The low prevalence rate of SB supplementation by UK and USA based team sport practitioners complements previous findings [12–14] and suggests that the translation of evidence from the laboratory to practice remains poor. Whilst contemporary studies have shown SB to improve team sport-specific exercise performance [2–4], it might be that low prevalence rates are explained by early work showing minimal benefits [32, 33], whereby results from studies take a while to feed through into the real-world. Most practitioners were aware of SB’s application as an ergogenic aid, and many believed that the supplement could improve performance. Typically, their awareness was attributed to reputable sources like scientific literature and higher education, rather than secondary information sources (e.g., social media/advertising). This contrasts with previous research showing that athletes’ understanding of nutritional supplementation strategies was from the internet or teammates [30, 31]. Our data also shows that the practitioners using SB opt for ingestion practices that follow evidence-based recommendations—most prescribe SB as a 0.2–0.3 g/kg BM dose, which is considered the optimal dosing strategy for eliciting benefits [34, 35]. Furthermore, supporting the majority of research, almost all suggested their reason for using SB was to improve performance, with SB widely given before matches, instead of its theoretical application during training and/or recovery that requires further investigation [36]. In light of these findings, it does not seem that inadequate surface level awareness limits the use of SB as an ergogenic aid in UK or USA team sports, and rather other reasons must be preventing widespread use of the supplement.

The most prominent barrier to the use of SB in team sports by our cohort of UK and USA based practitioners were concerns about GI side-effects. These have been documented after SB ingestion for years [32], therefore this finding is not surprising. Interestingly, only a few practitioners cited being unaware of benefits, or the belief that SB does not work, reaffirming our suggestion that low prevalence rates are not attributed to poor surface level awareness and understanding. That being said, it seems that negative connotations about GI distress following SB remain common in practitioners, even though extensive work has demonstrated traditional ingestion strategies (e.g., smaller dosing loads, split/progressive supplementation) to reduce side-effects [6, 17, 19]. Since GI discomfort after SB limits translation of scientific evidence to practice, it was expected that a higher proportion of practitioners would report using capsule ingestion strategies or cite alleviating side-effects as their main reason for selecting a certain SB administration approach. This suggests their understanding might be limited to surface level, such that they lack in depth scientific knowledge about strategies known to minimize side-effects. It is also possible, however, that the practitioners most concerned by GI side-effects are unwilling to trial alternative approaches—or simply omit using SB altogether—whereas those unaware of other approaches accept the risks. Whilst recent evidence suggests GI distress is inversely associated with performance [16], other studies have found benefits despite side-effects [17, 37], or no correlation between symptom severity and exercise performance [38]. Practitioners from the present study were not directly asked to explain why they consider GI side-effects to be a problem, but regardless of whether symptoms impair performance, they will likely disrupt preparation (e.g., warm-ups) and post-match (e.g., massages) routines. This may explain the limited prescription of SB in UK and USA team sports, and highlights educational needs in practitioners about SB administration approaches that minimize GI side-effects to ensure evidence-based practices are being adopted.

Another key theme that was highlighted from this study is that the awareness and practice of novel SB forms in UK and USA based team sport practitioners is limited, which provides a nuanced explanation for low prevalence rates. The emergence of novel SB forms like enteric-coated capsules, hydrogel mini-tablets, and topical muscle lotion within recent years will likely increase the real-world translation of SB, as they further alleviate concerns about GI side-effects demonstrated in our study to be the principal barrier to use. Since prevalence of the novel SB forms was anticipated to be low, practitioners were only asked which supplementation strategy they opted for most commonly, rather than if they used the three novel SB forms. Most of the practitioners who used SB with their athletes reported prescribing SB using traditional strategies (e.g., dissolved in solution), whilst a quarter of the whole cohort had not heard of any of the novel SB forms. It is therefore likely that their prevalence rate remains low, much like novel forms of other supplements (e.g., caffeinated chewing gum) [30]. Poor awareness and application of the novel SB forms compared to traditional ones might be attributed to several reasons. Firstly, these forms have only gained traction during the last five years [36], and consequently, it might take time for empirical evidence to translate into real-world practice. Secondly, research on enteric-coated capsules and hydrogel mini-tablets has focused on individual sports like running and cycling [21, 24, 25], meaning benefits for team sports remain largely unknown. Our group have conducted a body of work exploring the efficacy of a topical SB lotion in team sports, but equivocal findings have been reported for changes in HCO_3_^−^ concentration and performance [3, 4]. On the other hand, enteric-coated capsules and hydrogel mini-tablets are said to be effective SB delivery systems [22, 28], therefore placebo-controlled studies are now needed investigating their efficacy in team sports, as they may help overcome some of the barriers to use (e.g., GI side-effects) reported by practitioners from this study. Concomitantly, it would be prudent to examine the combined and comparative effects of SB to supplements like caffeine that are frequently used in team sports [11]. In an attempt to refine evidence-based practice based off insight gained from our study and existing literature gaps, it is recommended that future research explores the effectiveness of traditional and novel SB forms for improving performance and recovery in real-world team sport settings.

The strengths and limitations of this study must be acknowledged when interpreting outcomes. One of the strengths was our high completion rate, with 94% of practitioners finishing the survey. This can be attributed to careful planning and feedback gained from preliminary pilot testing, ensuring that wording was clear, and there were not too many questions. It is believed this prevented us missing any important data due to factors like respondent fatigue [39]. Most of our limitations can be explained by the challenges of conducting research in professional sport. Whilst recruiting practitioners rather than the athletes themselves was deemed necessary, it led to a relatively small sample size, with only 56% of practitioners working in professional sport. In some instances, particularly within collegiate sport, it is possible players took supplements outside of practitioners knowledge. Furthermore, prevalence rate of nutritional supplements is higher in professional athletes compared with other cohorts [14, 30], meaning a greater amount of practitioners might have reported prescribing SB if only professional team sports were surveyed. To maximize the proportion of practitioners involved with professional sport, the survey was distributed to practitioners working in UK and USA team sports. Country-specific comparisons were not made as this was outside of the scope of the current research question, and small sub-group sample sizes (UK: *n* = 40, USA: *n* = 26) did not permit sub-analyses. That being said, by collecting data across a broad geographical area, rather than a specific cohort (e.g., UK professional soccer clubs), it was not feasible for practitioners to complete the survey under supervision of a researcher. This might have influenced the accuracy of data, if some practitioners misinterpreted questions [10]. Also, prevalence rate for SB could be underestimated because of responses from practitioners in sports such as netball, American football and lacrosse, where research investigating the supplement is minimal, or lacking altogether. Lastly, our research team frequently distributed the survey to groups (e.g., practitioner WhatsApp chats) or individuals they personally knew. Although this ensured that the online survey reached its intended target—our respondents all reported ≥ 6 months experience working with team sports—it is possible that biases were introduced for the questions about the novel SB forms. Some of these practitioners would have been aware of our research [3, 4, 22], meaning they might have been more likely to report awareness and/or favourable opinions for hydrogel minitablets and topical muscle lotion. This could explain why a significant difference was found for the frequency of practitioners reporting that they would use novel SB forms.

## CONCLUSIONS

This study is the first to investigate practitioners’ prevalence and understanding of SB as an ergogenic strategy in team sports. It would appear that SB is not widely used by team sport practitioners based in UK and USA, despite most having good surface level awareness and understanding of its performance enhancing effects—primarily attributed to reputable information sources. The small proportion of practitioners prescribing SB supplementation as an ergogenic strategy typically adopt evidence-based practices by giving SB pre-match as a 0.2–0.3 g/kg BM dose. A large number of practitioners suggested GI side-effects as their main reason for not incorporating SB into nutritional regimes for team sport athletes. There appears to be a translational gap between science and the real-world, with practitioners unaware and/or unwilling to trial SB administration strategies that alleviate GI distress. Poor awareness of novel SB forms like hydrogel mini-tablets might be due to their recent emergence. Future research should aim to educate practitioners about SB administration strategies that alleviate GI side-effects and also compare the effectiveness of traditional and novel SB forms for improving performance and recovery in real-world team sport settings.
